# Revitalizing oral cancer research: Crispr-Cas9 technology the promise of genetic editing

**DOI:** 10.3389/fonc.2024.1383062

**Published:** 2024-06-10

**Authors:** Sowmya S. V., Dominic Augustine, Shazia Mushtaq, Hosam Ali Baeshen, Heba Ashi, Reem Nabil Hassan, Mohammed Alshahrani, Shankargouda Patil

**Affiliations:** ^1^ Department of Oral Pathology and Microbiology, Faculty of Dental Sciences, MS Ramaiah University of Applied Sciences, Bengaluru, Karnataka, India; ^2^ College of Applied Medical Sciences, Dental Health Department, King Saud University, Riyadh, Saudi Arabia; ^3^ Department of Orthodontics, Faculty of Dentistry, King Abdulziz University, Jeddah, Saudi Arabia; ^4^ Department of Dental Public Health, Faculty of Dentistry, King Abdulaziz University, Jeddah, Saudi Arabia; ^5^ Biological Sciences Department (Genome), Faculty of Sciences, King Abdul-Aziz University, Jeddah, Saudi Arabia; ^6^ Endodontic Department, Faculty of Dentistry, King Abdulaziz University, Jeddah, Saudi Arabia; ^7^ College of Dental Medicine, Roseman University of Health Sciences, South Jordan, UT, United States

**Keywords:** CRISPR-Cas9, gene editing, oral cancer, CRISPR-screens, genetic editing

## Abstract

This review presents an in-depth analysis of the immense potential of CRISPR-Cas9 technology in revolutionizing oral cancer research. It underscores the inherent limitations of conventional treatments while emphasizing the pressing need for groundbreaking approaches. The unparalleled capability of CRISPR-Cas9 to precisely target and modify specific genes involved in cancer progression heralds a new era in therapeutic intervention. Employing genome-wide CRISPR screens, vulnerabilities in oral cancer cells can be identified, thereby unravelling promising targets for therapeutic interventions. In the realm of oral cancer, the disruptive power of CRISPR-Cas9 manifests through its capacity to perturb genes that are intricately associated with drug resistance, consequently augmenting the efficacy of chemotherapy. To address the challenges that arise, this review diligently examines pertinent issues such as off-target effects, efficient delivery mechanisms, and the ethical considerations surrounding germline editing. Through precise gene editing, facilitated by CRISPR/Cas9, it becomes possible to overcome drug resistance by rectifying mutations, thereby enhancing the efficacy of personalized treatment strategies. This review delves into the prospects of CRISPR-Cas9, illuminating its potential applications in the domains of medicine, agriculture, and biotechnology. It is paramount to emphasize the necessity of ongoing research endeavors and the imperative to develop targeted therapies tailored specifically for oral cancer. By embracing this comprehensive overview, we can pave the way for ground-breaking treatments that instill renewed hope for enhanced outcomes in individuals afflicted by oral cancer.

## 1 Introduction

The emergence of CRISPR/Cas9 technology has triggered a profound revolution in the realm of molecular biology, facilitating precise and efficient genetic manipulation. This state-of-the-art tool empowers scientists with the capacity to meticulously modify genes within living cells, drawing inspiration from the intricate natural defense mechanisms employed by bacteria to shield themselves against viral threats (1). The term “CRISPR” denotes the distinctive pattern of DNA sequences observed in the bacterial genome, referred to as Clustered Regularly Interspaced Short Palindromic Repeats. The CRISPR/Cas9 system comprises two fundamental constituents: a guide RNA that directs the Cas9 enzyme to the precise genomic location where genetic editing is intended, and the Cas9 enzyme itself, which functions akin to molecular scissors, precisely cleaving the DNA at the targeted site identified by the guide RNA. This process activates the cell’s inherent repair mechanisms, leading to alterations in the genome with varying degrees of specificity and control (2).

The application of CRISPR/Cas9 technology in cancer treatment holds particularly promising prospects. Cancer manifests as a multifaceted genetic disorder stemming from mutations and other genetic anomalies, leading to uncontrolled cell growth and proliferation. While conventional treatments like chemotherapy and radiation therapy effectively eliminate cancer cells, they also inflict harm upon healthy cells and give rise to undesirable side effects. In contrast, CRISPR/Cas9 presents the potential to selectively target and modify cancer-associated genes, thereby yielding more effective and precise therapeutic interventions (2).

CRISPR/Cas9 finds extensive applications in biomedical research, encompassing investigations into gene function, the development of novel therapies for genetic diseases, and the creation of genetically engineered organisms. Within the realm of cancer research, CRISPR/Cas9 has been instrumental in elucidating the roles of cancer-related genes and advancing new cancer treatment modalities. Multiple studies have demonstrated the immense potential of this technology in both cancer diagnosis and treatment. Notably, it has exhibited remarkable efficacy in targeting and disrupting oncogenes, tumor suppressor genes, and other genes implicated in the initiation and progression of cancer. Recent research highlights the selective and efficient use of Non homologous end joining (NHEJ) CRISPR-mediated deletion of fusion oncogenes (FOs) in eliminating cancer cells, presenting a vital tool for basic research and holding potential as a therapeutic avenue (3). Furthermore, CRISPR/Cas9 has been employed to engineer T cells for immunotherapy, yielding promising outcomes in preclinical studies (4).

Despite recent strides, the utilization of CRISPR/Cas9 technology in the context of oral cancer remains relatively underexplored. Oral cancer represents a complex and heterogeneous form of head and neck cancer affecting various regions of the oral cavity, including the lips, tongue, and throat. It stands as a significant public health concern, ranking as the sixth most prevalent cancer worldwide, with over 377,000 new cases and 177,000 fatalities recorded in 2020 (5) Presently, the standard of care for oral cancer entails surgical interventions, radiation therapy, and chemotherapy; however, these treatments are often accompanied by substantial morbidity and mortality rates (6). Although CRISPR/Cas9 technology holds tremendous potential for advancing cancer research, its application in oral cancer is still in its nascent stage. Nevertheless, recent studies have shed light on the promising aspects of CRISPR/Cas9 in oral cancer, illustrating its ability to target specific genes involved in the development and progression of the disease. Notably, investigations have shown that CRISPR/Cas9 can effectively target the gene *MUC1* (Mucin short variant S1), which is frequently overexpressed in oral cancer cells. Targeting *MUC1* using CRISPR/Cas9 has demonstrated a significant reduction in the growth and proliferation of oral cancer cells (7).

The remarkable potential of CRISPR/Cas9 technology to overcome the limitations of existing oral cancer treatments necessitates thorough exploration of its applicability in this field. An expanding body of literature demonstrates the efficacy of CRISPR/Cas9 in targeting essential oncogenes and tumor suppressor genes both in laboratory settings and living organisms. The efficacy of CRISPR/Cas9 in targeting essential oncogenes and tumor suppressor genes has been demonstrated in both laboratory settings and living organisms. In a study by Jubair et al., systemic delivery of CRISPR/Cas9 targeting HPV oncogenes was effective in eliminating established tumors (8). Similarly, Gao et al. showed that CRISPR/Cas9 could selectively target the oncogenic KRAS G12S mutant allele, leading to efficient tumor regression. These findings suggest that CRISPR/Cas9 has the potential to be a powerful tool in cancer treatment (9). However, it is important to note that the technology is not without limitations, including off-target mutations and ethical considerations (10). Additionally, advancements in the delivery of CRISPR/Cas9 to oral cancer cells through diverse mechanisms like nanoparticles, liposomes, and viral vectors have been noteworthy (11–14).

**Figure 1:**
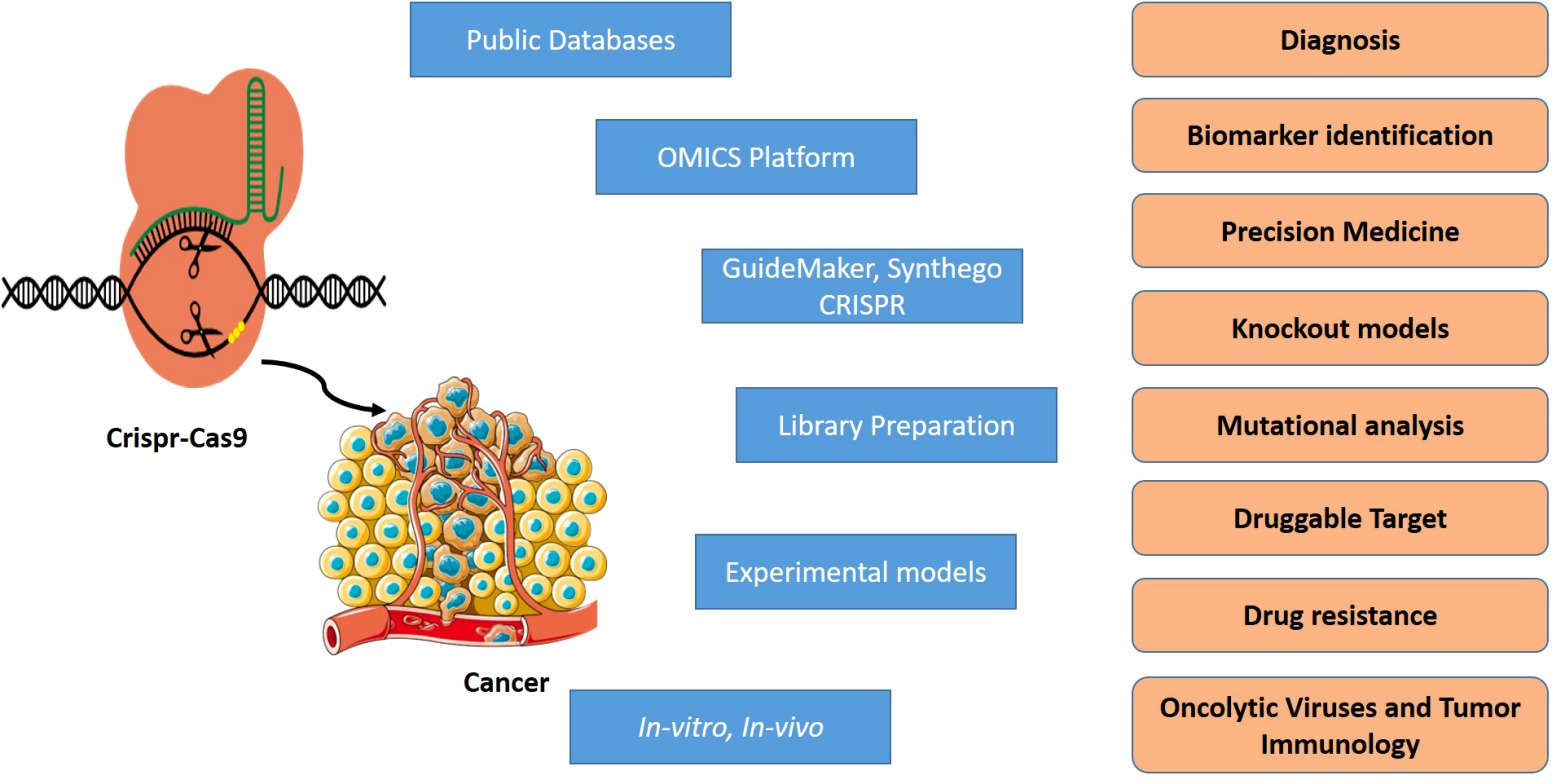
Application of CRISPR/Cas9 in cancer.

Hence, the objective of this review paper is to provide a comprehensive overview of the current state of knowledge concerning the utilization of CRISPR/Cas9 technology in the context of oral cancer. The specific focus is on the potential of CRISPR/Cas9 to target critical genes and pathways implicated in the development and progression of oral cancer. Furthermore, the challenges and opportunities associated with the implementation of this technology in this domain will be analyzed. The paper will also explore recent developments in the delivery of CRISPR/Cas9 to oral cancer cells and examine the prospects of combining it with other modalities such as immunotherapy (**Figure 1**). By compiling the latest research findings and insights, this paper aims to serve as a valuable resource for clinicians and researchers engaged in oral cancer research and those interested in the broader applications of CRISPR/Cas9 technology in the realm of cancer and beyond.

## 2 The potential of CRISPR/Cas9 in cancer treatment

CRISPR/Cas9 technology has revolutionized cancer research by offering a precise means of genome editing, allowing researchers to target and modify specific genes implicated in cancer development, thus paving the way for innovative approaches in cancer treatment (15). This section provides an overview of the current state of CRISPR/Cas9 applications across various cancer types and explores its role in the advancement of innovative therapeutic strategies.

### 2.1 Breast cancer

Recent research highlights the system’s effectiveness in identifying and validating genomic targets with tumorigenic potential, as well as its therapeutic applications (16, 17). Additionally, CRISPR/Cas9 shows promise in cancer immunotherapy, enhancing genome editing specificity and triggering a robust anti-tumor response (10). Moreover, its applications extend to breast cancer diagnostics, disease modeling, and treatment, including gene therapy development and malignant cell reprogramming (18). Collectively, these findings suggest the significant promise of CRISPR/Cas9 in breast cancer research and treatment.

### 2.2 Lung cancer

CRISPR/Cas9 technology has shown promise in the treatment of lung cancer, with applications including gene therapy, identification of target genes, and construction of animal tumor models (19). The first human trial using CRISPR/Cas9-modified cells for lung cancer treatment is set to take place in China, with a similar trial planned in the US (20). The technology is also being investigated as a potential tool in cancer immunotherapy, with ongoing animal models and human clinical trials (10).

### 2.3 Prostate cancer

Recent studies have highlighted the potential of CRISPR/Cas9 in prostate cancer treatment. A genetic screen for prostate cancer performed to date has contributed to the identification of biologically critical and clinically relevant target genes (21). CRISPR/Cas9 system accompanied by single- stranded oligonucleotide (ssODN) was shown to effectively repair the *TP53* 414delC gene region and inhibit the cell proliferation of PC-3 cells (22). Knockout of *NEAT1* gene using CRISPR/Cas9 technique caused significant changes in the expression of apoptosis-related genes in a way that the expression of P21, BCL2, and BIRC5 genes decreased (23). These studies collectively suggest that CRISPR/Cas9 holds potential for the treatment of prostate cancer.

### 2.4 Colorectal cancer

CRISPR/Cas9 technology has shown promise in the treatment of colorectal cancer, with potential applications in basic research, diagnosis, and personalized treatment (24). It has been used to correct causative mutations and enhance the immunological response in cancer immunotherapy, with ongoing clinical trials in immune cell modification (10). The technology has also been utilized for genetic screens, identification of druggable targets, and cancer gene therapy, with some applications in clinical trials (25). However, the technology’s limitations, such as off-target mutations and ethical concerns, need to be addressed (10).

### 2.5 Pancreatic cancer

CRISPR/Cas9 technology has shown promise in the treatment of pancreatic cancer, with potential applications in gene editing and targeted therapy (26). This technology, when combined with nanocarriers, has the potential to improve clinical outcomes for patients with pancreatic ductal adenocarcinoma (PDAC) (27). Furthermore, *CD73* deletion inhibited the ERK/STAT3 pathway and activated the E-cadherin pathway in human and murine cell lines (28). In preclinical studies, CRISPR/Cas9 has been used to induce pancreatic cancer in mice, demonstrating its potential for *in vivo* research (29).

### 2.6 Liver cancer

A range of clinical trials have explored the use of various treatments for liver cancer. Heo et al. found that JX-594, an oncolytic and immunotherapeutic vaccinia virus, demonstrated dose-related survival benefits in individuals with advanced hepatocellular carcinoma (30). Chen et al. reported promising results for camrelizumab, an anti-PD-1 antibody, in the treatment of primary liver cancer (31). Baskin-Bey et al. investigated the use of the pan-caspase inhibitor IDN-6556 in reducing liver cell apoptosis and injury during liver transplantation, with positive results (32). However, Newsome et al. found that granulocyte colony-stimulating factor (G-CSF) with or without haemopoietic stem-cell infusion did not improve liver dysfunction or fibrosis in patients with liver cirrhosis, and may be associated with increased adverse events (33). These studies highlight the potential of immunotherapies and oncolytic viruses in liver cancer treatment, but also the need for further research to confirm their efficacy and safety.

### 2.7 Ovarian cancer

The role of genes in ovarian cancer development has been investigated through the application of CRISPR/Cas9 technology. CRISPR/Cas9-mediated knockout of the *OC-2* negatively regulated the ovarian cancer progression possibly by apoptosis activation and angiogenesis inhibition, demonstrating its potential as a therapeutic tool (34). However, the safe and effective delivery of CRISPR/Cas-9 remains a challenge, with lipid-mediated delivery systems showing promise (35). Despite these challenges, CRISPR/Cas-9 is considered a powerful tool for the development of novel diagnostic approaches and therapeutic targets in ovarian cancer (36).

### 2.8 Leukemia

CRISPR/Cas9 technology has shown promise in the field of leukemia research, particularly in the study of chronic lymphocytic leukemia (CLL) and chronic myeloid leukemia (CML). Oshima et al. and Hernández-Sánchez et al. highlight the potential of CRISPR screening and genome-editing systems in understanding the role of mutated genes in leukemia pathogenesis and advancing functional studies of CLL (37). A CRISPR-Cas9 deletion system efficiently interrupts BCR/ABL1 oncogene in primary leukemic stem cells leading to the restoration of normal hematopoiesis, demonstrating potential therapeutic benefit (38). The CRISPR/Cas9 system is employed to investigate drug resistance in Acute Myeloid Leukemia (AML), enhance prognosis, and potentially move towards curing AML. Additionally, it aids in identifying new therapeutic targets, including the *FST* gene, lysine acetyltransferase *KAT7*, and *LSD1* (39). Furthermore, CRISPR-competent AML patient-derived xenograft models tractable for genome editing are valuable targets for translational value suggesting that CRISPR/Cas9 holds promise for both understanding the genetic basis of leukemia and potentially developing targeted therapies (40).

### 2.9 Melanoma

Recent studies have demonstrated the potential of CRISPR/Cas9 in treating melanoma. Wu et al. developed a light-inducible CRISPR-Cas9 system to inhibit the progression of melanoma cells by targeting the BRAF V600E mutation (41). This approach shows promise as a novel therapeutic strategy. On the contrary Giuliano et al. found that CRISPR/Cas9-induced mutations in the *MELK* gene did not result in a specific anti-proliferative phenotype, while Ercolano et al. study suggested that targeting the *PTGS2* gene could be a potential strategy for melanoma treatment (42, 43). These findings underscore the complexity of using CRISPR/Cas9 in cancer treatment and the need for further investigation.

### 2.10 Glioblastoma

CRISPR/Cas9 technology has shown promise in the treatment of glioblastoma multiforme (GBM), a highly aggressive brain tumor with poor prognosis (44). This gene-editing tool has been successfully applied in glioma stem cells, leading to efficient gene knockouts and the potential for genome-wide CRISPR screening (45). The use of lipid-based nano-delivery systems for *in vivo* CRISPR/Cas9 gene editing in brain cancer stem cells has also been explored, with promising results in disrupting the epidermal growth factor receptor variant III (EGFRvIII) mutation (46). Multiple studies have investigated the role of genes in GBM cell biology that can impact multiple survival pathways in cancers (47, 48). Furthermore, CRISPR-Cas9 gene-edited CAR T cells demonstrated enhanced activity in preclinical glioma models (49). These studies collectively highlight the potential of CRISPR/Cas9 in GBM treatment and the need for further research and clinical trials to validate its efficacy.

### 2.11 Oral cancer

Targeted treatments for cancer, which pinpoint specific genes essential for cancer cell survival, are limited for oral cancer. Understanding the genetic basis of oral cancer, particularly in Asian populations where most cases occur, remains a challenge. In the realm of CRISPR/Cas9 technology, research focused on oral cancer has gained momentum, aiming to elucidate the molecular mechanisms underlying oral carcinogenesis, identify potential therapeutic targets, and develop innovative treatment strategies. This technology has the potential to be a powerful tool in cancer therapy, with applications in gene editing, whole genome screening, and identifying cancer immunotherapy targets (50).

## 3 Genome-wide CRISPR screens

Genome-wide CRISPR screens have emerged as a potent methodology for elucidating gene function across diverse biological systems. These screens entail the utilization of CRISPR-Cas9 technology to generate a comprehensive repertoire of guide RNAs (gRNAs) targeting every gene within the genome. Subsequently, the gRNAs are introduced into cells, and the ensuing phenotypes are scrutinized to identify genes that play indispensable roles in specific biological processes (51).

Genome-wide CRISPR screens encompass large-scale experimental procedures employed to examine a population of mutant cells, with the goal of unveiling genes implicated phenotypes. The execution of genome-wide CRISPR screens entails a series of sequential steps. Initially, a CRISPR plasmid library is amplified, followed by the introduction of various genetically encoded perturbations into cell pools via pooled CRISPR screens. The targeted cells then proliferate under specific conditions, and the resultant phenotypes are subjected to analysis, leading to the identification of genes crucial for a given biological process (52). To generate libraries from genome-wide CRISPR screens, next-generation sequencing is often employed. These screens have shed light on intricate genetic interactions and have uncovered host factors crucial for viral replication.

The design of guide RNAs (gRNAs) to target every gene within the genome necessitates the employment of diverse strategies. A crucial aspect in any genome-wide CRISPR screen lies in the development of a set of unique gRNAs that selectively target the requisite gene set within the genome. The design of gRNAs for CRISPR genome editing entails the identification of a target site within the gene of interest, subsequently formulating a gRNA that can bind to said site. Numerous software tools, such as GuideMaker and the Synthego CRISPR Design Tool, facilitate the expeditious design of genome-wide gRNAs for any CRISPR-Cas enzyme, even in non-model organisms. Tools, including VISPR (VISualization of crisPR), a web-based interactive tool tailored for visualizing CRISPR screening experiments, facilitate the analysis of data generated by these screens (53). The distinct types of genome-wide CRISPR screens are detailed in **Table 1**.

**Table 1:** Functional Genomic Screens: Pooled vs. High-Content vs. Genome-Wide.

Pooled CRISPR screens	High-content CRISPR screens	Genome-wide CRISPR-Cas9 knockout screens
Perturbations are introduced in bulk and read out by guide RNA (gRNA)	Screens for multiple phenotypes simultaneously	Enables high-resolution, genome-wide functional genomic comparisons
Used to discover genes involved in a specific phenotype	Screens for genes involved in a specific phenotype	Enables high-resolution, genome-wide functional genomic comparisons
Large-scale experimental approach	Large-scale experimental approach	Large-scale experimental approach
Used to find a few needles in a haystack	Used to screen a population of mutant cells	Used to generate next-generation sequencing libraries
Example: Pooled CRISPR screens have been used to identify genes involved in cancer cell proliferation, migration, and invasion	Example: High-content CRISPR screens have been used to identify genes involved in cancer cell proliferation, migration, and invasion	Example: Genome-wide CRISPR-Cas9 knockout screens have been used to identify genes involved in cancer cell proliferation, migration, and invasion

### 3.1 Pooled CRISPR screens

Pooled CRISPR screens are a robust method for elucidating the essential genes in cancer cell survival. These screens involve the introduction of a library of single guide RNAs (sgRNAs) into cancer cells as a collective population, followed by the analysis of resulting phenotypes to identify indispensable genes (54). For instance, a recent study employed a CRISPR screen to identify essential genes in the survival of pancreatic cancer cells. Utilizing a custom CRISPR library targeting 18,009 genes, this study discovered a subset of genes, including those associated with DNA repair and cell cycle regulation, that are crucial for the survival of pancreatic cancer cells (55).

### 3.2 High-content CRISPR screens

High-content CRISPR screens represent a potent approach to identify genes that are pivotal for cancer cell survival. These screens offer remarkable possibilities to conduct mechanistic investigations on a large scale, employing advanced models like organoids and whole organisms, versatile perturbations such as gene activation and repression, diverse biological challenges, and data-rich readouts like single-cell sequencing and imaging (56). For instance, by performing a genome-wide CRISPR screen utilizing a T cell clone with a specific recognition mechanism for cancer cells independent of HLA, Holcomb et al. unveiled various genes crucial for tumor immunity, including those involved in antigen presentation and T-cell activation (57).

### 3.3 Genome-wide CRISPR-Cas9 knockout screens

Genome-wide CRISPR-Cas9 knockout screens hold significant potential in the identification of genes essential for the survival of cancer cells. These screens serve the purpose of elucidating the intricate genotype-phenotype relationship through the comprehensive disruption of gene expression on a genome-wide scale, enabling the examination of resulting phenotypic alterations. The technique leverages the CRISPR-Cas9 gene editing system in conjunction with libraries of single guide RNAs (sgRNAs) meticulously designed to target each gene within the genome (58). For instance, utilizing CRISPR-Cas9 in patient-derived GBM stem cells (GSCs), significant pathways for GBM growth are identified. Key genes such as those in the SOX transcription factor family, *SOCS3, USP8, DOT1L*, and protein ufmylation are implicated. Insights into temozolomide resistance suggest potential combination therapies. This functional approach uncovers genetic dependencies, enhancing comprehension of GBM treatment resistance (59).

### 3.4 Decoding genes: CRISPR screens in cancer research

The CRISPR/Cas9 system comprises two crucial components: a guide RNA that directs the Cas9 enzyme to the specific genomic location requiring genetic modification, and the Cas9 enzyme itself, acting as a molecular pair of scissors that cleaves DNA at the precise site guided by the RNA. This level of precision enables the cell’s natural repair mechanisms to make targeted and controlled alterations to the genome (60). Cancer, characterized by uncontrolled cell growth and proliferation, arises from genetic mutations and other genomic changes. While conventional therapies such as chemotherapy and radiation therapy effectively eliminate cancer cells, they also inflict damage to normal cells and entail adverse side effects. In contrast, CRISPR/Cas9 offers the ability to specifically target and modify cancer-related genes, thus providing safer and more precise therapeutic approaches (60).

In the field of biomedical research, CRISPR/Cas9 finds broad applications encompassing the study of gene function, the engineering of genetically modified organisms, and the exploration of innovative therapies for genetic disorders. In the context of cancer research, CRISPR/Cas9 has played a pivotal role in uncovering new avenues for cancer treatment and investigating the function of genes implicated in the disease. Multiple studies have showcased the remarkable diagnostic and therapeutic potential of this technology in the realm of cancer. It has demonstrated exceptional efficacy in targeting and disrupting oncogenes, tumor suppressor genes, and other genes involved in cancer development and progression. Moreover, CRISPR/Cas9 has shown promise in the engineering of T cells for immunotherapy, exhibiting encouraging outcomes in preclinical trials (61).

Oral cancer represents a complex and heterogeneous form of head and neck cancer affecting various structures within the oral cavity, such as the tongue, lips, and larynx. It is estimated that in 2020, over 377,000 new cases and 177,000 deaths worldwide will be attributed to oral cancer, ranking it as the sixth most prevalent cancer globally. Current standard treatments for oral cancer involve surgery, radiation therapy, and chemotherapy, all of which are associated with significant morbidity and mortality rates. Although CRISPR/Cas9 holds immense potential for advancing cancer research, its application to oral cancer is still in its nascent stages (62). Given the transformative potential of CRISPR/Cas9 technology in overcoming the limitations of existing oral cancer therapies, a comprehensive exploration of its utility in this context is imperative.

## 4 Unveiling the potential of CRISPR/Cas9 in oral cancer therapy

By conducting genome-wide CRISPR-Cas9 screens in oral squamous cell carcinoma (OSCC) cell lines, researchers can uncover genetic vulnerabilities that can be explored as potential targets for therapy. Furthermore, existing targeted therapies for identified genes may be repurposed for the treatment of this prevalent form of mouth cancer (62).

CRISPR-Cas9 technology also presents the opportunity to repair mutations or eliminate specific genes within tumors, offering a potential avenue for oral cancer treatment. It can be utilized to efficiently engineer oncolytic viruses and immune cells for therapeutic purposes in cancer. Notably, its capability to precisely modify genes extends beyond model organisms to humans, making it a valuable tool for therapeutic investigations. Additionally, it plays a crucial role in the development of comprehensive genomic libraries tailored to individual cancer patients (63). Therefore, the applications of CRISPR-Cas9 editing should prioritize efficacy, minimal undesirable outcomes, and long-term cellular impact. Identifying New Therapeutic Targets for Oral Squamous Cell Carcinoma Using CRISPR-Cas9 has been depicted in **Table 2**. The potential applications of CRISPR-Cas9 in addressing oral cancer include the following:

**Table 2:** Identifying New Therapeutic Targets for Oral Squamous Cell Carcinoma Using CRISPR-Cas9 Screens.

Evidence	Summary
Genome-wide CRISPR screens of oral squamous cell carcinoma	Several studies have conducted genome-wide CRISPR-Cas9 screens in oral squamous cell carcinoma (OSCC) cell lines to identify genetic vulnerabilities that can be explored as therapeutic targets. These studies have identified known and novel fitness genes that drive OSCC, especially among patients living in Asia. The data generated or analyzed during these studies are included in the manuscripts. Source data files for each figure and supplement have also been provided. Larger datasets of CRISPR screens, whole-exome sequencing, and RNA-sequencing output are available from Figshare.
Recombinant DNA reagent	The studies used recombinant DNA reagents such as Human Improved Genome-wide Knockout CRISPR Library v1 pooled library and pKLV2-EF1α-Cas9Bsd-W plasmid to conduct the genome-wide CRISPR-Cas9 screens
Cell line	The studies used various cell lines derived from oral squamous cell carcinoma, such as ORL-204, ORL-207, and ORL-214, to conduct the genome-wide CRISPR-Cas9 screens.
Targeted treatments	Uncovering the genes associated with oral squamous cell carcinoma opens the way for the development of new targeted treatments. Targeted therapies already exist for some of the genes identified in the studies, and it may be possible to repurpose them as a treatment for this widespread mouth cancer
Experimental details	The studies performed genome-wide CRISPR-Cas9 screens in triplicates and kept a minimum of 100x library coverage to maintain adequate representation of the sgRNA library. The statistical analysis methods used were described and justified, and raw statistical ranking data of the CRISPR screens were provided

### 4.1 CRISPR-Cas9 for investigating the mechanisms of tumorigenesis and development in oral cancer

CRISPR/Cas9 has emerged as a powerful tool for investigating the mechanisms of tumorigenesis and development in oral cancer. It has been used to identify cancer-specific vulnerabilities, generate organoid and mouse models of cancer, and track carcinogenic progression (64). In particular, CRISPR/Cas9 has been effective in identifying genes associated with oral cancer pathobiology, such as the *CD147*, and in exploring their potential as therapeutic targets (65). Furthermore, CRISPR/Cas9 has been used to establish a p75 neurotrophin receptor (p75NTR)-knockout cell line, which has led to a reduction in pro-tumorigenic behavior in oral cancer cells (66). These studies collectively demonstrate the potential of CRISPR/Cas9 in advancing our understanding of oral cancer and in developing targeted therapies.

### 4.2 CRISPR-Cas9 for identifying genetic vulnerabilities in oral squamous cell carcinoma cell lines

CRISPR-Cas9 can be utilized to identify genetic vulnerabilities in OSCC cell lines. By conducting genome-wide CRISPR-Cas9 screens in OSCC cell lines, researchers can identify genetic vulnerabilities that can be explored as potential therapeutic targets. This approach has already been employed to uncover new therapeutic targets for OSCC and develop targeted therapies for some of the genes identified in these studies (59).

### 4.3 CRISPR-Cas9 for discovering novel therapeutic targets for OSCC

CRISPR-Cas9 unveils novel therapeutic targets for OSCC, enabling the development of tailored therapies. CRISPR-Cas9 technology has been utilized to identify essential genes, druggable targets, and biomarkers for cancer research, aiding in understanding genotype-phenotype interactions and providing potential approaches for cancer treatment or diagnosis. Additionally, immune cells edited with CRISPR-Cas9 have been developed for cancer immunotherapies, with some already in phase I/II clinical trials (67). This approach has already led to the discovery of new therapeutic targets and the development of specific therapies (68).

### 4.4 CRISPR-Cas9 for editing genes in immune cells and oncolytic viruses for cancer therapeutic applications

CRISPR-Cas9 offers an effective method for precise gene modification in immune cells and oncolytic viruses, enhancing their potential for cancer therapeutic purposes. Through precise editing of genes in these entities, researchers can pioneer innovative therapies that specifically target cancer cells while safeguarding healthy ones. CRISPR/Cas9 has been extensively applied in cancer research for various purposes, and its effectiveness makes it a valuable tool for producing allogeneic CAR-T cells, potentially overcoming barriers in CAR-T cell therapy (69).

### 4.5 CRISPR-Cas9 for the establishment of comprehensive genomic libraries for cancer patients

CRISPR-Cas9 technology has revolutionized cancer research and therapy by enabling the establishment of comprehensive genomic libraries for cancer patients. Studies have highlighted the potential of CRISPR-Cas9 for identifying cancer-specific vulnerabilities and drug targets (70, 71). This is further supported by Pacini et al., who integrated large-scale CRISPR-Cas9 viability screens to identify genetic dependencies in cancer, and Michels et al., who used pooled CRISPR-Cas9 screening to identify tumor suppressors in human colon organoids (72, 73). These studies collectively underscore the potential of CRISPR-Cas9 for personalized cancer treatment through the establishment of comprehensive genomic libraries.

### 4.6 CRISPR-Cas9 for precision dentistry in the management of oral cancer

CRISPR-Cas9 presents a potent tool in precision dentistry, particularly for targeted oral cancer treatment. By precisely modifying genes in oral cancer cells, researchers can develop innovative therapies that selectively target malignancies while safeguarding healthy tissues. Moreover, harnessing CRISPR-Cas9’s capabilities in elucidating tumorigenesis mechanisms aids in identifying novel therapeutic targets and advancing treatment strategies (74). Notably, preclinical and clinical investigations have demonstrated promising outcomes with this approach in developing new cancer therapies. Additionally, recent studies underscore CRISPR-Cas9’s potential in identifying oral cancer-associated genes and its broader applications in precision dentistry, affirming its significant promise in oral cancer management (75).

## 5 Advancements and challenges of CRISPR/Cas9 technology

The remarkable advancements in CRISPR/Cas9 technology have ushered in a new era in molecular biology, offering unparalleled precision and efficiency in genome editing. However, alongside these advancements come significant challenges. Off-target effects, delivery methods, and ethical considerations remain major hurdles to overcome. Additionally, the complexity of the genome and the diverse range of applications require continuous refinement of CRISPR/Cas9 tools and techniques. Addressing these challenges will be crucial to fully harness the potential of CRISPR/Cas9 technology for diverse biomedical applications.

### 5.1 Advances in CRISPR/Cas9 technology

CRISPR/Cas9 technology has experienced rapid advancements since its initial discovery. Scientists have made significant modifications to enhance its efficiency, specificity, and accuracy. Notable progress includes the development of base editors, which enable precise modification of a single nucleotide in the DNA sequence without necessitating DNA cleavage. Additionally, the application of CRISPR/Cas9 for epigenetic editing has emerged, allowing for gene expression modulation without altering the DNA sequence. Moreover, researchers have devised novel delivery methods, such as nanoparticles and viral vectors, to enhance the efficiency and specificity of CRISPR/Cas9. These advancements have significantly expanded the potential applications of CRISPR/Cas9 technology across diverse domains, including medicine, agriculture, and biotechnology (50).

Integrating CRISPR-Cas9 with existing or emerging therapies, such as immunotherapy and personalized medicine, represents a promising frontier in the battle against oral cancer. By harnessing the precision of CRISPR-Cas9 gene editing alongside the targeted mechanisms of immunotherapy, researchers aim to develop synergistic treatment modalities that enhance the body’s immune response to cancer cells while precisely targeting genetic alterations driving tumor growth. This approach is employed in non-small cell lung cancer, and it is under clinical trials to investigate safety and feasibility (76, 77). Between 2017 and the beginning of 2022, six CAR-T immunotherapy drugs received approval from both the U.S. Food and Drug Administration (FDA) and the European Commission (EC) for B-cell lymphoma, Mantle cell lymphoma and B-cell precursor acute lymphoblastic leukemia, and relapsed refractory multiple myeloma (78). Furthermore, personalized medicine approaches, guided by genomic profiling, enable tailored treatments that consider individual variations in tumor biology, treatment response, and potential side effects. However, challenges such as off-target effects and delivery efficiency remain significant hurdles that need to be addressed for the successful translation of CRISPR-Cas9-based therapies into clinical practice.

### 5.2 Preclinical applications of CRISPR/Cas9 technology

CRISPR/Cas9 technology has shown significant potential in preclinical applications, particularly in genome editing and gene therapy. Multiple studies have underscored the versatility and efficiency of CRISPR/Cas9 in these areas, noting its use in controlling transcription, modifying epigenomes, and conducting genome-wide screens (79, 80). Targeted genome engineering enables precise modification of genetic information for studying gene function and disease pathology. The CRISPR/Cas9 applications in biomedical discoveries have been broad and successful, despite some off-target effects (81). Furthermore, CRISPR/Cas9 has played a pivotal role in the engineering of cells for immunotherapy, such as chimeric antigen receptor T-cell (CAR-T) therapy for cancer treatment (82). CRISPR/Cas9 technology combined with stem cells is a powerful tool for generating various cell types for disease modeling, drug screening, toxicology, and targeted therapies. The system has been used for genetic modification of stem cells, leading to the differentiation of specific cell types for functional analysis and potential clinical transplantation. Recent advancements in CRISPR/Cas9 technology have expanded the possibilities for stem cell research and therapeutic applications (83). These preclinical endeavors have demonstrated the tremendous potential of CRISPR/Cas9 technology in the development of innovative therapies for a wide spectrum of diseases.

### 5.3 Current challenges in CRISPR/Cas9 technology

CRISPR/Cas9 technology has made remarkable strides in gene therapy research, disease modeling, and precision medicine. However, there remain several challenges that necessitate attention before its safe and effective implementation in clinical settings. One of the foremost challenges pertains to the potential for off-target effects, which may result in unintended genomic mutations. Off-target effects arise when Cas9 acts upon non-targeted genomic sites, inducing cleavages that can yield adverse outcomes. This concern is of paramount significance as it has the potential to inflict enduring harm upon the genome, leading to the development of malignancies (84).

Another challenge lies in the delivery of CRISPR/Cas9 to target cells, which can prove arduous in certain cases. Delivery to target cells stands as a pivotal step in the gene editing process, necessitating an efficient, safe, and specific delivery system. Various delivery methods exist, encompassing viral and non-viral vectors. However, each method harbors its own limitations, and the selection of an appropriate delivery approach hinges upon the specific application at hand (85).

The landscape of programmable nucleases, including meganucleases (MNs), zinc finger nucleases (ZFNs), transcription activator-like effector nucleases (TALENs), and clustered regularly interspaced short palindromic repeats-Cas (CRISPR-Cas), has also contributed to genome editing from research to clinical and industrial domains. Each technology exhibits unique modes of action impacting their applicability across the genome and is subject to distinct testing conditions. While CRISPR-Cas9 leads the field owing to its versatility and rapid adoption, it is not without limitations. Contextual considerations dictate the selection of the most suitable approach for genetic modification within a given organism. For instance, the precision of meganucleases may suffice for high-precision applications where efficiency is not paramount, while ZFNs and TALENs, despite their efficiency, face challenges in retargeting. CRISPR-Cas stands out for its versatility, allowing for straightforward retargeting via guide RNA modification and compatibility with various delivery methods. Safety considerations, particularly in clinical applications, require thorough assessment of off-target effects and other potential risks (86).

Recent advancements in drug delivery systems have focused on overcoming challenges such as off-target effects and improving delivery mechanisms. Zhao et al. and Singh et al. both highlight the importance of tissue-specific drug delivery and the use of computational models to design and deliver drugs across biological barriers (87, 88). Nanomaterial-based drug delivery systems have also shown promise in addressing these challenges, with Chatterjee et al. discussing their applications and advancements, including stimulus-responsive and multifunctional nanocarriers (89). There is emphasis on the potential of nanoplatforms for targeted stimuli-responsive drug delivery, particularly in cancer therapy (90). These studies collectively underscore the potential of targeted drug delivery systems in minimizing off-target effects and improving therapeutic efficacy. Further, CRISPR/Cas9 off-target effects can be predicted online *in-silico* tools (91). These tools primarily rely on sgRNA sequences, potentially overlooking intracellular nuclear milieu. Strategies like enhancing Cas9 mutants such as eSpCas9 and SpCas9-HF1, exploring Cas9 homologs with rarer PAM sequences, and improving delivery methods significantly impact CRISPR/Cas9 gene editing fidelity. For *in vivo* gene therapy, CRISPR/Cas9 editing of somatic cells is a major application. To ensure safety, it’s crucial to directly measure off-target effects in tissues and living organisms. Techniques like Discover-seq and GUIDE-tag offer promising avenues for such assessments. Notwithstanding these challenges, CRISPR/Cas9 has showcased its potential utility in treating various diseases, including cancer, and holds promise for the development of novel therapies targeting hereditary conditions.

### 5.4 Implications of CRISPR/Cas9 technology

The emergence of CRISPR/Cas9 technology has engendered significant ethical and societal implications. Chief among the concerns is the potential for inadvertent consequences, encompassing off-target effects and unintended mutations. Another apprehension revolves around the possibility of unethical utilization of the technology, such as human germline editing. Furthermore, equitable distribution of the benefits arising from CRISPR/Cas9 technology, particularly in developing countries, raises concerns. These implications necessitate careful consideration as the technology continues to advance (92).

### 5.5 Safety and ethical concerns in CRISPR/Cas9 technology

In addition to technical advancements, a comprehensive review of CRISPR-Cas9’s medical deployment necessitates a thorough examination of its ethical implications and societal impact. Ethical considerations surrounding germline editing pose fundamental questions regarding the manipulation of heritable traits in future generations, raising concerns related to consent, autonomy, and human dignity. Safety concerns also extend to the possibility of off-target effects and the long-term ramifications of gene editing upon the genome (93). Establishing robust ethical and regulatory frameworks is imperative to navigate these complex ethical landscapes and ensure the responsible application of CRISPR-Cas9 technology.

### 5.6 CRISPR/Cas9 technology in the treatment of medical conditions

Preclinical studies have demonstrated the tremendous potential of CRISPR/Cas9 technology in treating diverse genetic disorders, including sickle cell anemia, cystic fibrosis, and Huntington’s disease. The system has also been instrumental in engineering cells for immunotherapy, exemplified by the utilization of CAR-T cells for cancer treatment. Furthermore, CRISPR/Cas9 can be employed to develop novel diagnostic tools for infectious diseases (82). The scope of potential applications in medical condition treatment using CRISPR/Cas9 technology is vast, and researchers continue to explore innovative avenues. Nonetheless, several challenges demand resolution before CRISPR/Cas9 can be safely and effectively employed in clinical settings. One notable challenge pertains to the possibility of off-target effects, which may induce unintended mutations in the genome. Delivery of CRISPR/Cas9 to target cells poses another challenge, particularly in certain scenarios. Additionally, the employment of CRISPR/Cas9 raises safety and ethical concerns, particularly concerning human germline editing (94). Addressing these challenges is imperative to ensure the secure and efficacious application of CRISPR/Cas9 technology in clinical settings. Notwithstanding these challenges, the potential of CRISPR/Cas9 technology in the development of innovative therapies for a wide array of diseases is immense, and researchers are diligently striving to overcome these obstacles to deliver these therapies to patients.

## 6 CRISPR/Cas9 gene editing for overcoming drug resistance in oral cancer

Drug resistance poses a significant challenge in the treatment of oral cancer, hindering the efficacy of conventional therapies and limiting patient outcomes. Comprehending the development of drug resistance and devising innovative strategies to overcome it is pivotal in advancing oral cancer treatment. Drug resistance in oral cancer typically arises from a spectrum of mechanisms, including genetic mutations, altered drug metabolism, augmented DNA repair, and activation of cellular survival pathways. These mechanisms collectively enable cancer cells to evade the cytotoxic effects of chemotherapy drugs, resulting in treatment failure and disease progression. Consequently, researchers have sought diverse approaches to impede drug resistance in cancer, with the utilization of CRISPR/Cas9 gene editing technology emerging as one of the most promising strategies (95).

Genome-wide CRISPR/Cas9 screening in oral cancer research has potential to unveile drug resistance genes enriched within a given pathway. However, screening faces challenges due to cell heterogeneity and factors like sgRNA library construction. Single-cell CRISPR/Cas9 screening offers comprehensive insight into genetic alterations but at a higher cost. Meta-analysis of screening models could construct a gene-related network elucidating drug resistance. Using CRISPR/Cas9, researchers targeted CD147 in cal27 cells to explore its role in doxorubicin resistance in oral cancer. Knocking out CD147 led to decreased proliferation, invasion, and matrix metalloproteinase expression. Nude mice models confirmed reduced doxorubicin resistance and proliferation in the knockout cell line, highlighting CD147 as a promising target for overcoming drug resistance in oral cancer (65). CRISPR/Cas9 has proven effective in investigating the functionalities of cancer-related genes, establish animal models bearing tumors, and elucidate drug targets, substantially expanding our comprehension of cancer genomics (96). In the context of oral squamous cell carcinoma (OSCC), researchers have undertaken genome-wide CRISPR-Cas9 screenings across 21 cell lines, thereby identifying genetic vulnerabilities that could serve as potential therapeutic targets. This endeavor has led to the identification of both known and novel fitness genes that drive oral squamous cell carcinoma, particularly among patients residing in Asia, where over half of the yearly cases are diagnosed. By systematically inactivating genes in these cell lines, researchers successfully identified 918 genes crucial to the survival of OSCC cells. The screenings successfully identified known and novel fitness genes that drive oral squamous cell carcinoma, a prevalent form of mouth cancer. This identification paves the way for the development of targeted treatments, as targeted therapies already exist for some of the genes discovered in this study. Repurposing these therapies may offer a viable treatment option for this widespread malignancy (97).

To address drug resistance in oral cancer, CRISPR/Cas9 can be utilized in a similar manner. By identifying key genes involved in oral cancer drug resistance, such as those associated with drug metabolism, DNA damage repair, or cell survival pathways, researchers can employ CRISPR/Cas9 to disrupt or modify these genes. This disruption or modification can enhance the efficacy of chemotherapy drugs and restore sensitivity to treatment. The application of CRISPR/Cas9 in overcoming drug resistance in oral cancer holds tremendous potential. However, further research and preclinical studies are necessary to identify the specific genes and mechanisms involved in oral cancer drug resistance. Additionally, optimizing delivery methods and ensuring the safety and efficiency of CRISPR/Cas9 in clinical settings are essential steps towards its successful implementation.

## 7 Future applications of CRISPR/Cas9 technology

CRISPR/Cas9 technology has a wide range of potential applications, including genome engineering, antimicrobial development, disease modeling, and food science (98–101). It can be used to edit DNA, modulate gene expression, and create precision antimicrobials (98). In disease animal models, it has been used for gene function studies, genetic breeding, and disease treatment (99). In human disease modeling, it has been applied for gene editing, regulation of gene expression, and high-throughput screening (100). In food science, it has potential for pathogen typing, vaccination, and the development of enhanced foods (101). CRISPR/Cas9 technology allows for precise genome editing in eukaryotic cells, revolutionizing molecular biology and offering a promising strategy for anti-viral therapy by targeting and editing viral DNA in infected cells (102). Despite these promising applications, there are still challenges and limitations that need to be addressed.

Nevertheless, several challenges necessitate resolution before the safe and effective implementation of CRISPR/Cas9 in clinical settings. A primary concern pertains to the potential for off-target effects, which may result in unintended mutations within the genome. Off-target effects arise when Cas9 acts on unintended genomic sites, leading to cleavages that could yield adverse consequences. This poses a significant apprehension, as it could induce lasting damage to the genome, potentially culminating in the development of cancer or other diseases. CRISPR/Cas9 technology has demonstrated significant promise in the treatment of diverse cancers, including oral cancer. The identification of genetic vulnerabilities in OSCC cells through comprehensive genome-wide CRISPR/Cas9 screenings lays the groundwork for the development of targeted treatments for oral cancer. Nonetheless, several challenges, such as off-target effects, effective delivery to target cells, and safety and ethical considerations, must be effectively addressed before the widespread implementation of CRISPR/Cas9 in clinical settings.

## 8 Conclusion

CRISPR/Cas9 technology holds tremendous potential across various domains, encompassing medicine, agriculture, and biotechnology. Its revolutionary capabilities are reshaping scientific research and presenting novel prospects for addressing genetic diseases, cancer, and infectious diseases. In the field of medicine, CRISPR/Cas9 demonstrates remarkable efficacy in precisely targeting and rectifying genetic mutations, laying the groundwork for innovative therapeutic interventions. By leveraging CRISPR/Cas9, it becomes possible to engineer oncolytic viruses and immune cells, opening up exciting avenues for cancer treatment. Moreover, the construction of comprehensive genomic libraries facilitated by CRISPR/Cas9 provides valuable insights into cancer genomics, aiding in the customization of treatment approaches.

Within cancer research, CRISPR/Cas9 has emerged as a powerful instrument for unravelling the mechanisms governing tumorigenesis and development. Through genome editing and the precise targeting of mutations, researchers can gain crucial insights into the drivers behind cancer initiation and progression. Comprehensive screenings employing CRISPR/Cas9 enable the identification of genetic vulnerabilities within oral cancer cells, offering valuable guidance for the development of targeted therapeutic strategies tailored to specific cancer types. These breakthroughs pave the way for personalized medicine and the potential repurposing of existing targeted therapies to combat prevalent oral cancer and other malignancies.

Notwithstanding the immense potential of CRISPR/Cas9, it is crucial to address several challenges before its widespread implementation in clinical settings. A primary concern is the potential for off-target effects, whereby unintended mutations in the genome may yield adverse outcomes, including the emergence of cancer. Ensuring the safe and efficient delivery of CRISPR/Cas9 to target cells represents another critical hurdle, necessitating the development of precise and dependable delivery methods. Additionally, ethical considerations associated with human germline editing raise profound questions regarding the unintended consequences and long-term impacts of modifying the DNA of future generations.

Overcoming these challenges is imperative to harnessing the full potential of CRISPR/Cas9 technology. Continued research and preclinical investigations are indispensable for deepening our understanding of the specific genes and mechanisms underlying drug resistance and tumorigenesis. Moreover, optimizing delivery methods while enhancing the safety and efficiency of CRISPR/Cas9 in clinical applications are pivotal steps towards realizing its transformative impact. While significant obstacles lie ahead, persistent exploration and refinement of CRISPR/Cas9 will undoubtedly propel us towards a future where precise genetic editing becomes a reality, unlocking new frontiers in scientific discovery and improving the lives of individuals worldwide.

## Author contributions

SS: Conceptualization, Data curation, Formal analysis, Resources, Visualization, Writing – original draft. DA: Conceptualization, Data curation, Formal analysis, Resources, Visualization, Writing – original draft. SM: Conceptualization, Data curation, Formal analysis, Writing – original draft. HB: Conceptualization, Data curation, Formal analysis, Writing – original draft. HA: Conceptualization, Data curation, Formal analysis, Writing – review & editing. RH: Data curation, Formal analysis, Validation, Writing – review & editing. MA: Data curation, Formal analysis, Validation, Writing – review & editing. SP: Project administration, Resources, Supervision, Validation, Visualization, Writing – review & editing.
